# Biceps femoris calcific tendinitis as a rare cause of lateral knee pain: a case report

**DOI:** 10.1097/MS9.0000000000003021

**Published:** 2025-03-27

**Authors:** Shivaji Karki, Sushil Pokhrel, Karuna Khadka

**Affiliations:** a Department of Orthopedics, Health Service Office, Rukum West, Rukum West, Nepal; b Department of Internal Medicine, Pokhara Academy of Health Science, Pokhara, Kaski, Nepal

**Keywords:** biceps femoris, calcific tendinitis, knee pain

## Abstract

**Introduction:**

Calcific tendinitis is a common cause of periarticular pain, though it rarely affects the distal biceps femoris tendon, leading to lateral knee pain. This case report highlights a rare instance of biceps femoris calcific tendinitis and its successful conservative management.

**Case presentation:**

A 40-year-old female presented with a 1-week history of sharp lateral knee pain, which worsened with movement but persisted at rest. There was no history of trauma or systemic illness. On examination, tenderness was localized to the lateral knee, specifically over the fibular head and biceps femoris tendon. Blood tests were unremarkable. Radiographs and ultrasound revealed multifocal calcifications at the fibular head. Although MRI was recommended, it was not feasible in this resource-limited setting. The patient was initially treated conservatively with NSAIDs and physiotherapy but did not respond. She subsequently underwent ultrasound-guided barbotage and corticosteroid injection, resulting in the complete resolution of symptoms within 1 week.

**Discussion:**

Calcific tendinitis is caused by the deposition of calcium hydroxyapatite crystals in periarticular tendons, leading to inflammation and pain. Biceps femoris calcific tendinitis is rare, and diagnosis can be made using cost-effective imaging modalities such as radiography and ultrasound. Conservative management, including NSAIDs, physiotherapy, and ultrasound-guided barbotage, is the preferred first-line treatment. In this case, the combination of barbotage and physiotherapy led to complete symptom relief.

**Conclusion:**

Biceps femoris calcific tendinitis, though rare, should be considered in cases of lateral knee pain. Early diagnosis and conservative treatment can result in rapid symptom relief.

## Introduction

Calcific tendinitis is a common cause of periarticular pain, most commonly involving the rotator cuff tendon. Lessor common sites are Achilles tendon, longus colli, and gluteus maximus. The distal tendon of the biceps femoris is an exceptionally rare site for involvement, causing sharp, persistent lateral knee pain that worsens with activity^[1]^. It is managed similarly to other causes of calcific tendinitis, using both conservative and surgical approaches^[2]^. Through an extensive literature search, we found only two previously reported cases of biceps femoris calcific tendinitis. Here, we present a case to demonstrate that this previously rarely described cause of knee pain can be managed conservatively with satisfactory resolution. The work has been reported in line with the SCARE 2023 criteria^[3]^.

**Highlights**

- **Rare condition**: Biceps femoris calcific tendinitis is an extremely rare cause of lateral knee pain, with only two prior cases reported in the literature.
- **Case presentation**: A 40-year-old female presented with 1 week of sharp lateral knee pain, worsening with movement and persistent at rest.
- **Diagnostic approach**: Diagnosis was based on plain radiography and ultrasound, revealing multifocal calcifications at the fibular head. MRI was advised but not feasible in this setting.
- **Treatment and outcome**: Conservative management with NSAIDs and physiotherapy was initially unsuccessful. However, ultrasound-guided barbotage and corticosteroid injection resulted in complete symptom resolution within 1 week.
- **Significance**: The case underscores the importance of considering biceps femoris calcific tendinitis in the differential diagnosis of lateral knee pain and demonstrates effective conservative treatment strategies.

## Case report

**Figure 1.**
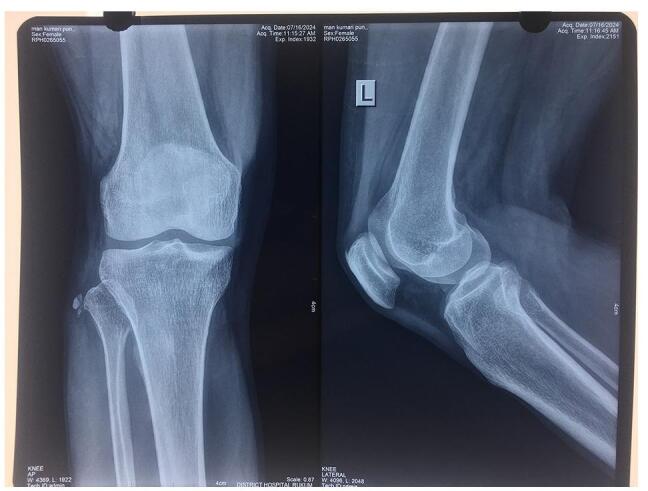
Antero-posterior and lateral knee X-rays demonstrate multifocal calcium deposits at the lateral aspect of the proximal fibula.

**Figure 2.**
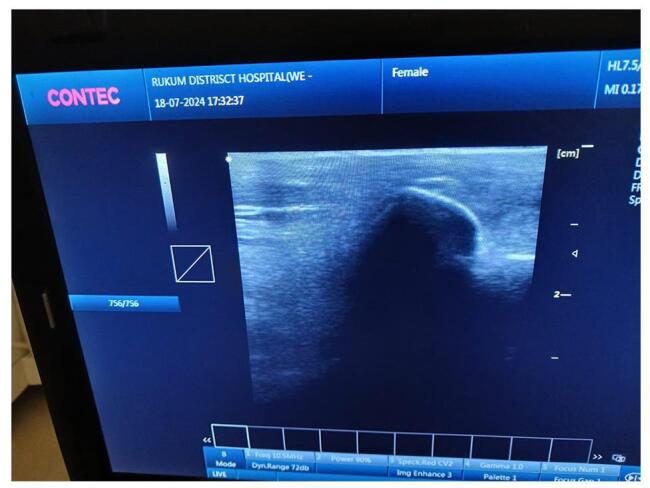
USG image of left knee showing calcifications on lateral aspect.

**Figure 3.**
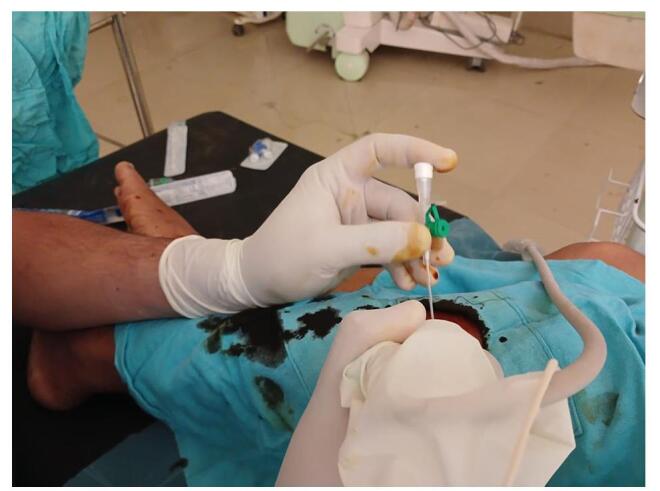
USG-guided barbotage.

A 40-year-old female presented to our orthopedic outpatient department (OPD) at the remote district hospital of Nepal with a 1-week history of lateral knee pain. The pain had gradually worsened and was sharp in nature. It began after routine household activities, with worsening during movement, though it persisted at rest. She had no history of fever, trauma, or prior knee problems, and her medical history was unremarkable. On examination, her body temperature was normal, and there were no signs or symptoms of systemic illness. Local inspection showed no swelling or erythema. Tenderness was noted on palpation of the lateral aspect of the knee, with point tenderness over the head of the fibula and the biceps femoris tendon. The range of motion was normal, but flexion resistance induced pain in the lateral knee. The patient’s gait was normal, and no crepitus was noted on movement. The varus stress test was positive, while the Lachman test, McMurray test, anterior drawer test, posterior drawer test, and valgus stress test were all negative. These findings suggested localized tendinopathy as a cause of pain. Blood tests showed normal inflammatory markers (C-reactive protein: negative, erythrocyte sedimentation rate: 8 mm/h, white blood cell count: 9200 mm^−3^). A plain radiograph revealed a multifocal dense opacity along the lateral aspect of fibular head (Fig. 1). Ultrasound also showed foci of calcification in the lateral aspect of the fibular head (Fig. 2). MRI was advised; however, we did not have it at our level of hospital, and the patient could not afford to visit a higher center for the procedure. A diagnosis of calcific tendinitis of the biceps femoris was made based on X-ray and ultrasound findings. She was initially started on conservative management with NSAIDs and physiotherapy. However, she did not respond well. She subsequently underwent ultrasound-guided barbotage of the calcium deposits (Fig. 3) and received a peri-tendinous corticosteroid injection, which provided complete symptomatic relief. She was followed up after 1 week, during which NSAIDs and physiotherapy were continued. At that time, she reported being pain-free, and upon a 1-month follow-up, she remained asymptomatic, with no tenderness noted.

## Discussion

Calcific tendinitis results from the deposition of calcium hydroxyapatite crystals in periarticular muscular attachments. It causes inflammation, necrosis, and loss of tissue structure. Its causes are unknown; however, it has been associated with trauma, diabetes and chronic kidney diseases, and metabolic causes such as hyperparathyroidism and other hypercalcemic states^[4,5]^. Our patient, however, did not have significant medical issues. Calcific deposits around the knee involve vastus lateralis^[6]^, popliteus tendons^[7]^, and the lateral collateral ligament^[8]^. When calcification is involved around the knee, symptoms include pain and tenderness at the site of attachment. Popliteal calcific tendinitis may present with acute pain and spasms of the popliteus and may mimic a “locked knee”^[7]^. Biceps femoris calcific tendonitis in our situation presented with pain at the tendon insertion site; proximal and lateral aspect of the fibular head with normal range of motion. Radiography is the most practical and cost-effective modality for evaluating calcific tendinitis. It is useful for not only detecting calcium deposits but also assessing their extent, delineation, and density. Typically, there is no radiographic evidence of degenerative joint disease. Ultrasound (USG) is also valuable in visualizing the calcification and determining its extent. Magnetic resonance imaging (MRI) is an important tool in evaluating calcific tendinitis when earlier investigations yield inconclusive results^[9]^. However, in resource-limited settings like ours, plain radiography and/or USG can reliably be used to diagnose biceps femoris calcific tendinitis. The biceps femoris, together with the semitendinosus and semimembranosus, forms the hamstring muscles. It originates from two heads: the long head from the ischial tuberosity and the short head from the lateral part of the linea aspera. These two heads converge in the mid-thigh and insert into the head of the fibula, splitting around the lateral collateral ligament. As a result, calcific tendinitis of the biceps femoris is typically visible on the lateral aspect of the fibular head in radiographic imaging. Conservative management is always the first-line treatment for calcific tendinitis. This includes non-steroidal anti-inflammatory drugs (NSAIDs), steroid injections, physiotherapy, ultrasound (USG)-guided barbotage, and extracorporeal shock wave therapy^[6,10,11]^. As our patient did not respond well to NSAIDs, she had to undergo ultrasound-guided barbotage, followed by steroid injection. USG-guided barbotage combined with steroid injection has been shown to be superior to steroid injection alone. Surgical removal of calcium deposits should only be considered after 6 months of unsuccessful conservative management^[10]^. With the combination of ultrasound-guided barbotage and 1 week of physiotherapy, our patient experienced complete resolution of symptoms within the 1-week time frame.

## Conclusion

Biceps femoris calcific tendinitis, though rare, can be a cause of lateral knee pain. It can be effectively diagnosed with plain radiography. This case report may aid in understanding the condition, facilitating early diagnosis, and guiding appropriate management. Conservative management, including ultrasound-guided barbotage with steroid injection, can be effective even in resource-limited, remote settings.

## Data Availability

Publicly available.

## Research registration unique identifying number (UIN)

N/A.
